# Case Report: Transcatheter closure of ventricular septal defect using a Starway Neo Occluder via a single-access approach: report of two cases

**DOI:** 10.3389/fcvm.2026.1774886

**Published:** 2026-02-24

**Authors:** Changjing Huang, Nan Cai, Youqian Li, Weike Wu, Jingfeng Liu, Haifeng Hong, Guodong Zhang, Zhihui Hu, Wei Zhong

**Affiliations:** 1 Center for Cardiovascular Diseases, Meizhou People’s Hospital, Meizhou Academy of Medical Sciences, Meizhou, China; 2 Department of Ultrasound, Meizhou People’s Hospital, Meizhou Academy of Medical Sciences, Meizhou, China; 3 Department of Interventional Medicine, Meizhou People’s Hospital, Meizhou Academy of Medical Sciences, Meizhou, China; 4 GuangDong Engineering Technological Research Center of Molecular Diagnosis in Cardiovascular Diseases, Meizhou, China; 5 Meizhou Key Laboratory of Cardiovascular Translational Medicine, Meizhou, China

**Keywords:** Starway Neo occluder, ventricular septal defect, interventional closure, right brachial artery, transcatheter single-access technique

## Abstract

**Introduction:**

Ventricular septal defect (VSD) is among the most prevalent congenital heart diseases, accounting for approximately 20%–30% of cases. Mainstay treatments encompass interventional closure and surgical repair. The conventional interventional approach necessitates puncturing both the femoral artery and vein to establish an arteriovenous circuit for device delivery. While markedly less invasive than open-heart surgery, this method carries inherent risks, including sheath compression challenges, conduction block, injury to valvular chordae tendineae, and vascular complications.

**Case description:**

To enhance procedural safety while maintaining efficacy, our center pioneered a transcatheter single-access closure technique for VSD, performed under fluoroscopic and echocardiographic guidance in two patients. This “single” strategy proved successful in both instances. The procedures were well-tolerated, with patients achieving ambulation on the same day, experiencing stable perioperative periods, and demonstrating favorable early recovery outcomes. Immediate and one-month follow-up transthoracic echocardiography confirmed stable device position, with no evidence of residual shunt or new-onset valvular regurgitation.

**Conclusion:**

These case reports preliminarily indicate that the transcatheter single-access VSD closure technique is technically feasible and demonstrates a favorable early safety profile. While limited in scale, the successful outcomes in these two cases suggest significant promise for broader clinical adoption. However, further validation through larger-scale studies and long-term follow-up is required to confirm its sustained efficacy and safety.

## 1 Introduction

Ventricular septal defect (VSD) is the most common congenital heart disease, with an estimated incidence of 20%–30%. While conventional transcatheter closure is a well-established treatment that enables many patients to avoid open surgery, certain limitations such as technical challenges during the procedure and a higher risk of postoperative complications have drawn increasing attention. To address these issues, Professor Zhong Wei from our cardiovascular center developed the Single-access technique (“Single technique”). By innovating the surgical approach and utilizing newly designed devices, this method simplifies the procedure, facilitates operation, and promotes faster recovery, thereby enhancing overall procedural safety. In this report, we describe two patients with perimembranous VSDs measuring 2.8 mm and 3.0 mm, respectively, located ≥2 mm from the aortic valve. Both defects were successfully closed using the Single technique.

## 2 Case description

### 2.1 Case 1

A 72-year-old female presented to our hospital with “recurrent dizziness and fatigue for 1 year”.

#### 2.1.1 Preoperative evaluation

**Figure 1:**
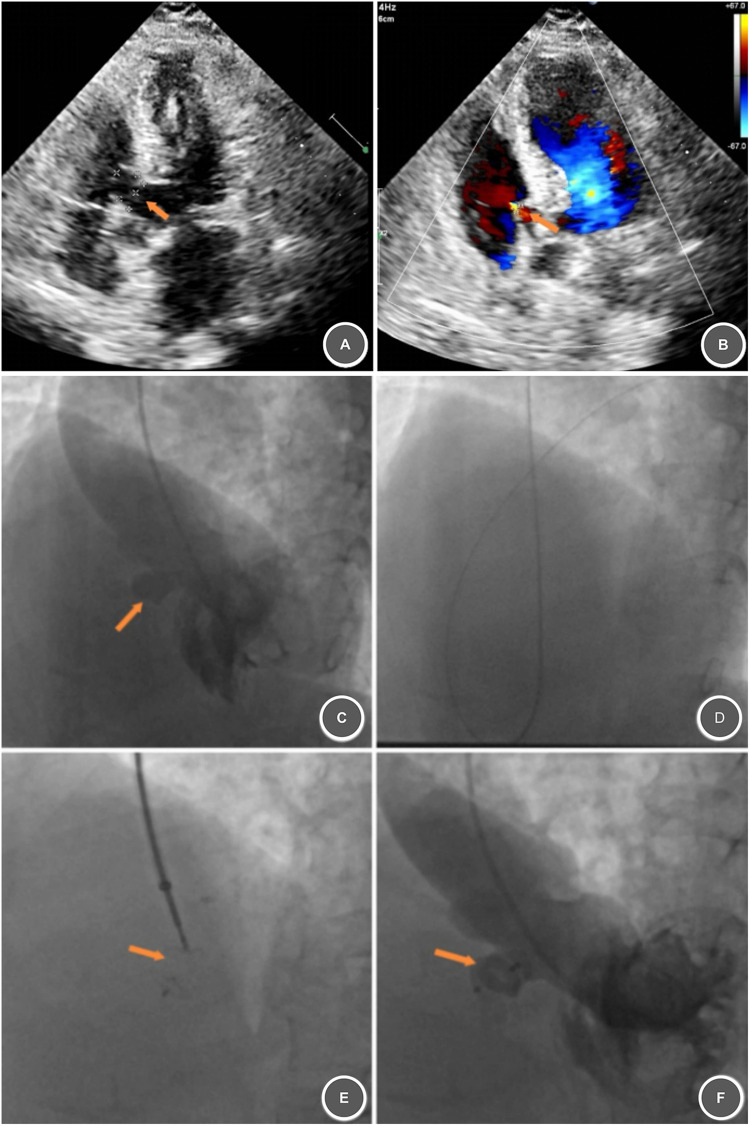
Preoperative and Intraoperative imaging of a perimembranous VSD with aneurysm formation treated by transcatheter closure. **(A)** Membranous aneurysm formation, measuring approximately 12 × 11 mm (arrow). **(B)** Multiple sieve-like perforations on the right ventricular surface, with the largest measuring approximately 3 mm in width (arrow). **(C)** Left ventricular angiography confirming VSD location and size (∼3.5 mm, arrow). **(D)** Established treatment tract. **(E)** Deployed ventricular septal occluder (arrow). **(F)** Post-deployment angiography showing stable occluder position and morphology (arrow).

- **Color flow jet across the septum**: Grade 3–4/6 systolic murmur at the left sternal border (3rd–4th intercostal spaces).
- **Transthoracic Echocardiography**: Ventricular septal defect with membranous aneurysm formation (Figures 1A,B), measuring approximately 12 × 11 mm; multiple sieve-like perforations on the right ventricular side (larger defect ∼3 mm wide); mild mitral and tricuspid regurgitation.
- **Past Medical History**: No hypertension, coronary heart disease, cerebral infarction, or pulmonary hypertension.

According to the Guidelines for Percutaneous Interventional Treatment of Common Congenital Heart Diseases (2021 Edition) (1), transcatheter VSD closure was performed after obtaining informed consent.

#### 2.1.2 Surgical procedure

Preoperatively, based on a profound understanding of cardiac/vascular anatomy, thorough knowledge of the novel domestic VSD occluder, and consideration of the patient's advanced age (to reduce risks of traditional dual access), we proposed the Transcatheter Single-Access Ventricular Septal Defect Closure Technique (abbreviated as Single Technique) for the first time. The specific steps were as follows (Figures 1C–F): (1) Under local anesthesia, puncture the right brachial artery. (2) Use a 5F pigtail catheter for left ventriculography to confirm VSD location and size (Figure 1C). (3) Under fluoroscopic and echocardiographic guidance, establish a “right brachial artery → left ventricle → VSD → right ventricle” tract with a guidewire (Figure 1D). (4) Select a 7 mm symmetrical, flexible Starway Neo Occluder (with retractable waist) for closure (Figure 1E). (5) Post-occluder release: Echocardiography confirmed stable occluder position/morphology, no aortic valve regurgitation. Repeat left ventriculography (10 min later) showed no residual shunt (Figure 1F).

- **Procedure Metrics**: Total time = 40 min; fluoroscopy time = 9 min.
- **Intraoperative Monitoring**: Sinus rhythm was observed on continuous electrocardiographic monitoring throughout the operation, with no abnormalities noted.

#### 2.1.3 Postoperative recovery & follow-up

**Figure 2:**
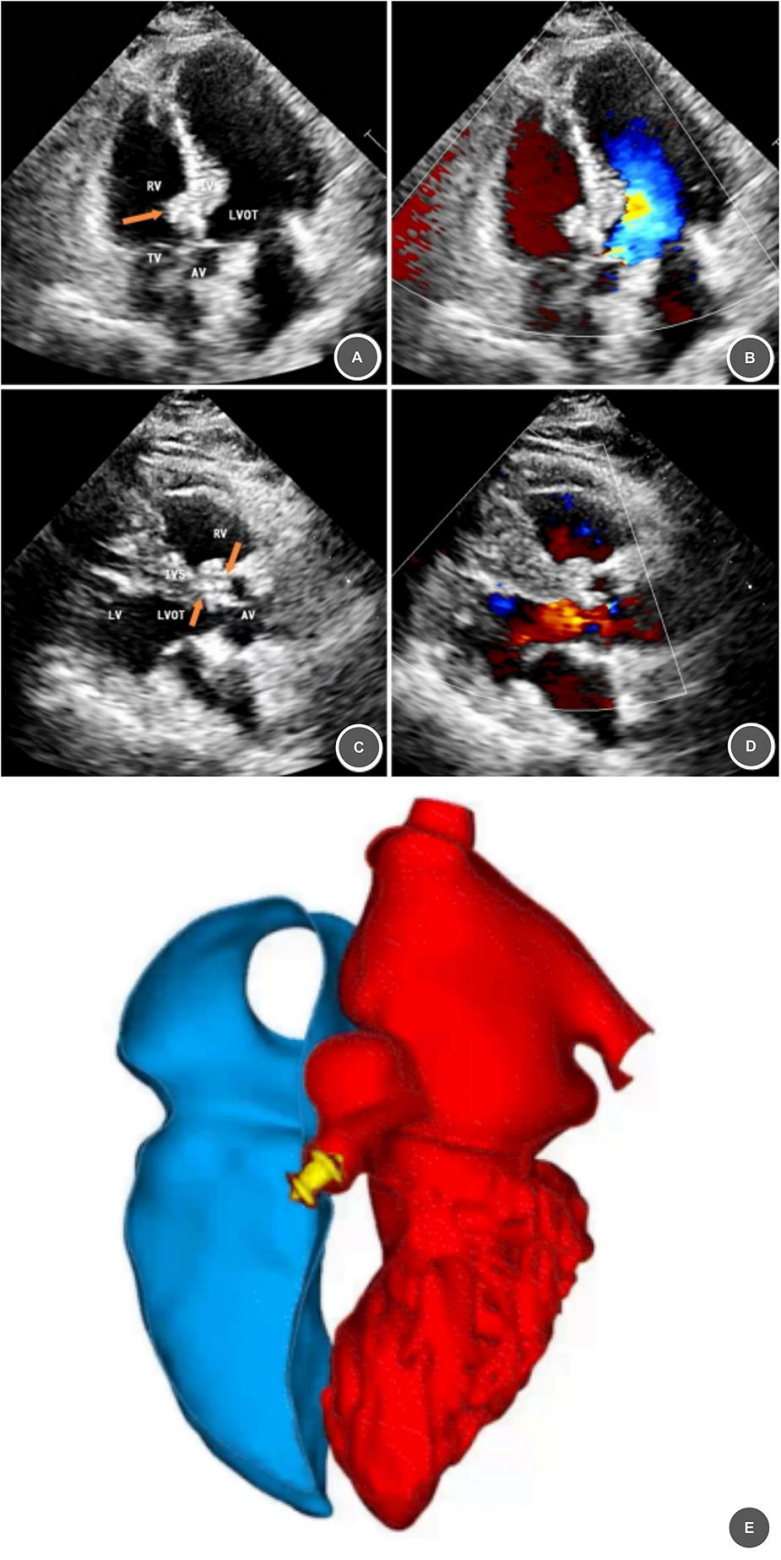
One–month postoperative follow, up imaging after VSD closure. **(A–D)** One-month postoperative echocardiography demonstrating a well-positioned and stable occluder with no significant residual shunt (arrow). **(E)** One-month postoperative cardiac CT (cardiac cavity reconstruction and rendering) showing localized metallic density in the ventricular septum, confirming stable device morphology and position.

- **Immediate Postoperative**: The patient received aspirin enteric-coated tablets for antiplatelet therapy; ambulated immediately without impairment to daily activities.
- **1-Month Follow-Up**:
  ○ Cardiac auscultation revealed a regular rhythm with no systolic murmur.
  ○ Transthoracic Echocardiography: Stable occluder position, no residual shunt or new valvular regurgitation (Figures 2A–D).
  ○ Cardiac CT: Localized metallic density in the ventricular septum (fixed morphology/position; Figure 2E).
  ○ Complications: None (device- or procedure-related).

### 2.2 Case 2

A 12-year-old male was admitted to the ward due to “discovery of ventricular septal defect 1 week ago”.

#### 2.2.1 Preoperative evaluation

**Figure 3:**
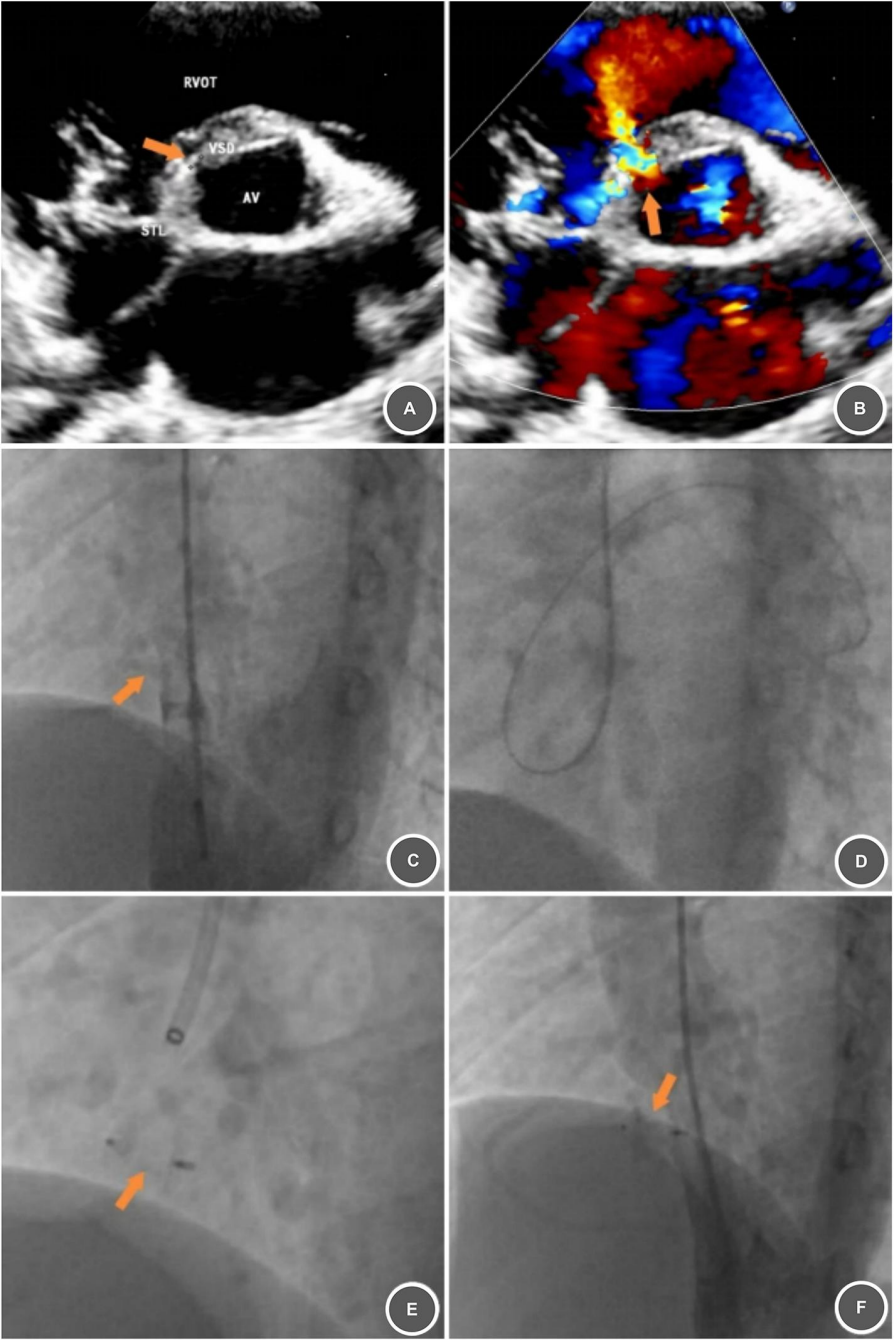
Preoperative and intraoperative imaging of a second perimembranous VSD case treated with a Starway Neo Occluder. **(A,B)** Preoperative echocardiography demonstrating a membranous ventricular septal defect (∼2.8 mm) with a visible color trans-septal jet (arrow). **(C)** Left ventricular angiography confirming VSD location and size (∼3.0 mm, arrow). **(D)** Established treatment tract. **(E)** Deployed Starway Neo Occluder (arrow). **(F)** Post-deployment angiography showing stable occluder position and morphology (arrow).

- **Cardiac Auscultation**: Grade 3/6 pathological systolic murmur at the left sternal border (3rd–4th intercostal spaces).
- **Transthoracic Echocardiography**: Perimembranous ventricular septal defect (∼2.8 mm in size) with a left-to-right shunt across the defect (Figures 3A,B); normal valve morphology/echo/motion.
- **Past Medical History**: No comorbidities.

#### 2.2.2 Surgical procedure

Imaging confirmed the defect was perimembranous, small in diameter, and compatible with a 5F system. To facilitate early return to school, the Single Technique was planned after obtaining family informed consent. The steps were as follows: (1) Under local anesthesia, place a 5F arterial sheath via the right brachial artery. (2) Administer heparin (100 U/kg) intravenously. (3) Left ventriculography confirmed VSD (∼3.0 mm; Figure 3C). (4) Establish the treatment tract (Figure 3D), then deploy a 6 mm domestic ventricular septal occluder (with retractable waist; Figure 3E). (6) Post-procedure angiography: Stable occluder position/morphology (Figure 3F).

**Figure 4:**
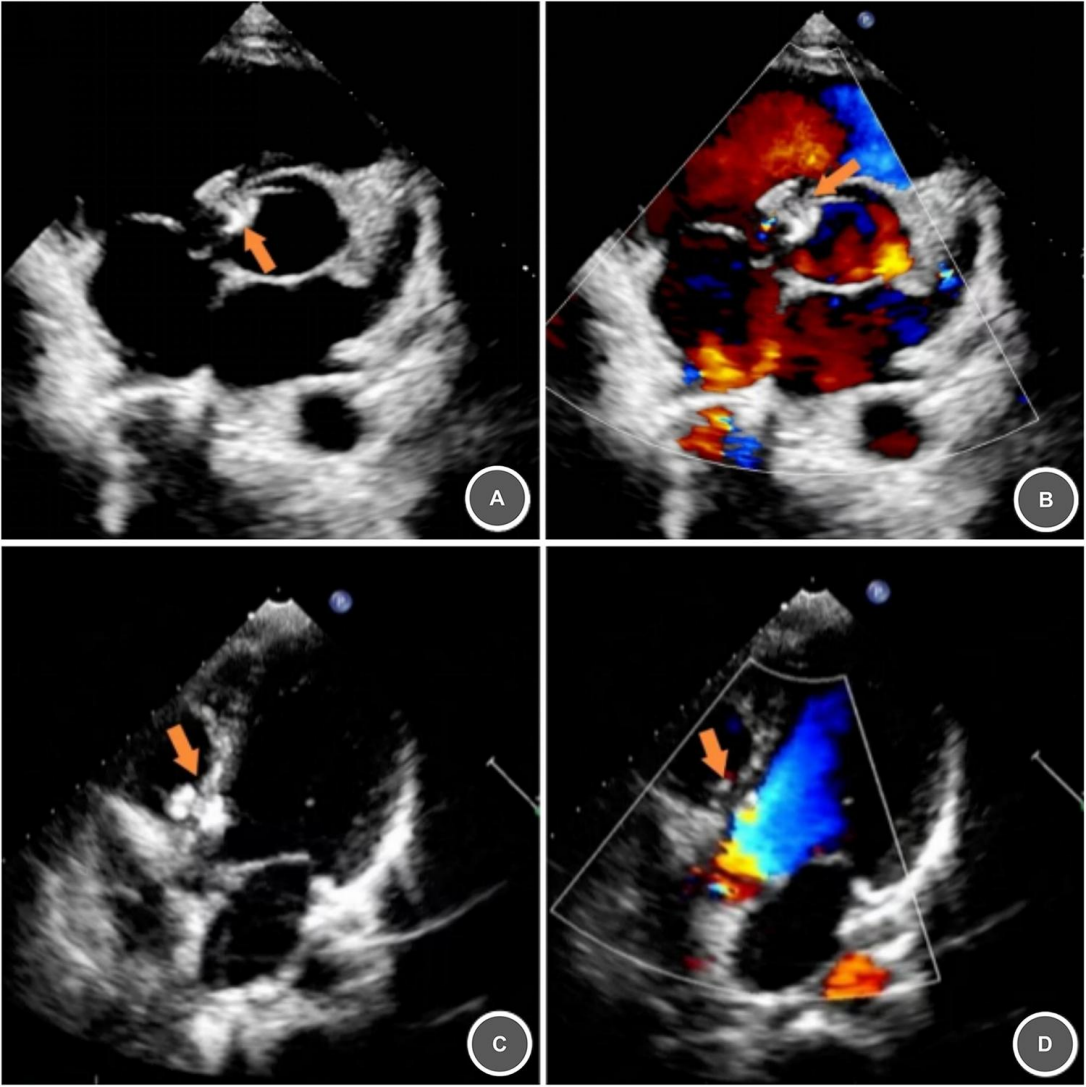
Immediate and one–month postoperative echocardiographic follow, up after VSD closure. **(A,B)** Postoperative echocardiography demonstrating a well-positioned and stable occluder with no significant residual shunt (arrow). **(C,D)** One-month postoperative echocardiography shows the occluder is well-positioned and stable, with no significant residual shunt (arrow).

- **Procedure Metrics**: Total time = 47 min; fluoroscopy time = 7 min.
- **Intraoperative Monitoring**: Continuous intraoperative ECG monitoring revealed normal sinus rhythm. The patient ambulated independently from the operating table. Post-procedural echocardiography demonstrated appropriate occluder position, absence of residual shunt, and normal valvular function (Figures 4A,B).

#### 2.2.3 Postoperative recovery & follow-up

- 1-Month Follow-Up:
- Cardiac auscultation revealed a regular rhythm and no systolic murmur.
- Transthoracic echocardiography showed stable occluder position and no significant residual shunt (Figures 4C,D); no device- or procedure-related complications.

## 3 Discussion

### 3.1 Background of transcatheter VSD closure

Transcatheter closure has become the mainstream treatment for perimembranous ventricular septal defects (pmVSD). Since Lock et al. (2) first reported transcatheter closure of pmVSD via the femoral arteriovenous double-access approach in 1988, the technique has advanced rapidly. However, the traditional “double-access, long-circuit” procedures still carry risks (e.g., vascular injury, tricuspid valve chordae tendineae injury, atrioventricular block) due to complex pathways and high guidewire tension (1, 3).

Recent retrograde transfemoral artery single-access techniques have shortened procedures and reduced costs (4, 5), but early studies relied on rigid Amplatzer occluders (fixed waist design), which risked valve injury, embolization, or residual shunt and were only suitable for patients with ideal anatomy (4–6). Later studies [e.g., Zhao et al. (7) using Amplatzer duct occluder 2; Piccinelli et al. (8) using Amplatzer Vascular Plug-II] showed soft, symmetrical occluders improved outcomes—but device specifications and sheath size limited application in adults or patients with small vascular access.

### 3.2 Advantages of the single technique & Starway Neo Occluder

The occluder used in this study was the novel Neo VSD occluder, which incorporates several innovative design features compared to conventional devices:

1. **Materials and Weaving:**
  It is woven using an improved technique, resulting in softer discs that reduce myocardial compression and lower the risk of conduction block.
2. **Structural Design:**
  ○ **Retractable waist:** Adapts to defects of varying lengths and morphologies.
  ○ **Waist occlusion membrane:** Ensures complete closure and minimizes residual shunt.
  • **Models and Delivery:**
    Available in 12 waist diameters (4–18 mm). All models are delivered via a **5–7F sheath**, minimizing vascular injury and making them suitable for trans-brachial single access approaches.

The two cases of perimembranous ventricular septal defect (VSD) reported in this study both involved small defects (measuring 2.8 mm and 3.0 mm, respectively) with relatively intact rims and no obvious fenestrations or tunnel-like morphology. These anatomical features facilitated precise positioning and device deployment via the retrograde approach. Furthermore, the distance from the superior edge of the defects to the aortic valve was ≥2 mm in both cases, with no concomitant aortic valve prolapse or regurgitation, providing the necessary anatomical space for the safe implantation of a Starway Neo Occluder. In contrast, complex VSDs characterized by larger size, thin or deficient rims, or proximity to the aortic valve often require conventional dual-access or more intricate interventional strategies. Therefore, the cases presented here represent an exploratory application of the single-access technique, conducted under stringent anatomical selection criteria.

The two successful cases demonstrate four key advantages of the Single Technique:

1. **Innovative Operative Pathway.** This technique abandons the conventional femoral arteriovenous double puncture and instead establishes a short operative tract through a single arterial access. This approach eliminates the traditional “sheath compression” effect and avoids high-tension guidewire traction on the tricuspid valve chordae tendineae—a known risk factor for new tricuspid regurgitation in dual-access procedures. Furthermore, compared to the transradial approach, single-access transbrachial VSD closure offers distinct advantages in procedural trauma control and patient experience. The radial artery is limited by its smaller caliber in certain patients, higher propensity for spasm, and narrower range of compatible sheath sizes. In contrast, the brachial artery provides a relatively larger lumen, facilitating smoother passage of delivery sheaths and improved device maneuverability. Its straighter anatomical course enhances catheter stability, while its superficial location allows for easier compression hemostasis post-procedure. These features support earlier postoperative mobilization, improve patient comfort, and may contribute to shorter hospital stays, collectively positioning the transbrachial route as a more minimally invasive option for single-access retrograde VSD closure (9).
2. **Process Simplification & Radiation Protection.** Omitting femoral vein puncture and complex sheath manipulation (10) shortens procedure time (40–50 min) and fluoroscopy time (7–9 min), reducing radiation exposure for staff and patients—aligning with minimally invasive and radiation safety principles (11).
3. **Access Expansion & Rapid Postoperative Recovery.** The 5F sheath makes the brachial approach suitable for children (≥4 years old) and adults with limited vascular access. Immediate postoperative ambulation improves patient experience and avoids immobilization-related restrictions of femoral artery techniques.
4. **Biomechanical Optimization of the Occluder.** The soft, symmetrical structure and retractable waist reduce clamping force on surrounding tissues, lowering atrioventricular block risk. Indirect evidence from a similar CE-certified domestic device (12) shows high closure rates (98.7%), low residual shunt rates (1.3%), and low conduction injury rates (0.8%)—supporting the safety of the Starway Neo Occluder used here.

### 3.3 Limitations

1. The duration of follow-up was limited; therefore, the long-term efficacy remains to be evaluated through extended monitoring.
2. Anatomical Restriction: If the defect margin is ≤2 mm from the aortic valve, aortic valve motion may affect device placement (requires further anatomical assessment).
3. Pediatric Application: Suitability for pediatric pmVSD closure and operational standardization need additional research.
4. Long-Term Efficacy: Multi-center, large-sample studies are required to validate long-term outcomes and standardized procedures.

## 4 Conclusion

The successful trans-brachial single-access VSD closure in these two cases demonstrates the technique's potential to simplify operations, expand access, and optimize outcomes—particularly for pmVSD patients with appropriate anatomy. To date, our center has successfully treated 8 VSD patients with this technique (satisfactory closure results), supporting its clinical promotion.

## Data Availability

The original contributions presented in the study are included in the article/Supplementary Material, further inquiries can be directed to the corresponding author.
